# Pushing Stoichiometries of Lithium-Rich Layered Oxides Beyond Their Limits

**DOI:** 10.1021/acsaem.1c03396

**Published:** 2022-02-11

**Authors:** Arcangelo Celeste, Rosaria Brescia, Giorgia Greco, Piero Torelli, Silvia Mauri, Laura Silvestri, Vittorio Pellegrini, Sergio Brutti

**Affiliations:** † Dipartimento di Chimica e Chimica Industriale, Università degli Studi di Genova, via Dodecaneso 31, 16146 Genova, Italy; ‡ Graphene Labs, Istituto Italiano di Tecnologia, via Morego 30, 16163 Genova, Italy; § Electron Microscopy Facility, Istituto Italiano di Tecnologia, via Morego 30, 16163 Genova, Italy; ∥ Dipartimento di Chimica, Università di Roma La Sapienza, p.le Aldo Moro 5, 00185 Roma, Italy; ⊥ Laboratorio TASC, Istituto Officina dei Materiali (IOM)−CNR, Area Science Park, S.S.14, km 163.5, I-34149 Trieste, Italy; # Dipartimento di Fisica, University of Trieste, via A. Valerio 2, 34127 Trieste, Italy; ¶ Dipartimento di Tecnologie Energetiche e Fonti Rinnovabili, ENEA C.R. Casaccia, via Anguillarese 301, 00123 Roma, Italy; ∇ BeDimensional Spa, via Torrentesecca 3d, 16163 Genova, Italy; ○ GISEL—Centro di Riferimento Nazionale per i Sistemi di Accumulo Elettrochimico di Energia, INSTM, via G. Giusti, 50121 Firenze, Italy; ⧫ ISC-CNR OUS Sapienza, Via dei Tarquini, 00185 Roma, Italy

**Keywords:** Li-ion battery, positive electrodes, transition-metal oxides, layered materials, Co-poor

## Abstract

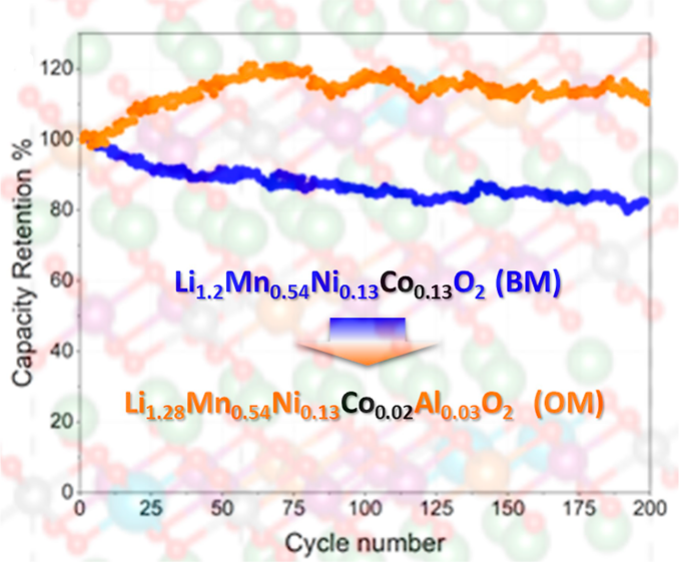

Lithium-rich layered oxides (LRLOs) are opening unexplored frontiers for high-capacity/high-voltage positive electrodes in Li-ion batteries (LIBs) to meet the challenges of green and safe transportation as well as cheap and sustainable stationary energy storage from renewable sources. LRLOs exploit the extra lithiation provided by the Li_1.2_TM_0.8_O_2_ stoichiometries (TM = a blend of transition metals with a moderate cobalt content) achievable by a layered structure to disclose specific capacities beyond 200–250 mA h g^–1^ and working potentials in the 3.4–3.8 V range versus Li. Here, we demonstrate an innovative paradigm to extend the LRLO concept. We have balanced the substitution of cobalt in the transition-metal layer of the lattice with aluminum and lithium, pushing the composition of LRLO to unexplored stoichiometries, that is, Li_1.2+*x*_(Mn,Ni,Co,Al)_0.8–*x*_O_2−δ_. The fine tuning of the composition of the metal blend results in an optimized layered material, that is, Li_1.28_Mn_0.54_Ni_0.13_Co_0.02_Al_0.03_O_2−δ_, with outstanding electrochemical performance in full LIBs, improved environmental benignity, and reduced manufacturing costs compared to the state-of-the-art.

## Introduction

In the last decades, a wide variety of new electrode materials have been developed and demonstrated for innovative lithium-ion battery (LIB) formulations to push performance beyond the state-of-the-art.^1,2^ Indeed, the massive change in the societal needs from the beginning of the century to present is leading to a remarkable energetic demand increase, beyond short-term fluctuations, calling for more efficient, cheap, and more sustainable energy storage technologies such as batteries.^3^ High-capacity positive electrode materials are the key component to increase the energy and power densities of any future LIB formulation.

Lithium-rich layered oxides (LRLOs) are a well-known wide family of mixed metal oxides with general formula Li_1+*x*_M_1–*x*_O_2_, where M is a blend of transition metals, typically containing manganese and cobalt. These materials can supply capacities in the order of 200–250 mA h g^–1^ and operating potentials in the range 3.4–3.8 V versus Li, overcoming conventional layered oxide cathode materials.^4−6^ The peculiar crystal structure of LRLOs, made up of a coexistence of two lattices partially sharing crystal symmetries [i.e., the α-NaFeO_2_ rhombohedral (hR12) and the Li_2_MnO_3_ monoclinic (mC24) lattices],^7−9^ allows for a redox activity originated by oxidation/reduction of transition-metal ions and above 4.2 V by the partially reversible O_2_^2–/^O_2_^4–^ couple on the anion sublattices.^10,11^ This last lithium exchange mechanism leads, on charge, to irreversible molecular oxygen release at high potentials.^12^ Therefore, the accumulation of oxygen vacancies and subsequent rearrangements on the cation sublattice promote lattice distortions upon cycling resulting in the long term to wide structural transition into a spinel lattice.^13,14^ These structural changes lead to a monotonic mean redox potential decay and capacity fading.^15,16^ Nevertheless, the search of innovative LRLO with tailored transition-metal blends and doping is rushing,^17,18^ aimed at reducing the cobalt content of this class of materials but, at the same time, ameliorating performances. Cobalt reduction has been identified by EU and DOE^19,20^ as a major driver to improve the environmental benignity of batteries and the sustainability of the overall production–consumption–recycling lifecycle. Furthermore, cobalt is a strategic commodity traded with rising prices on the international markets, and therefore, its minimization in a battery formulation substantially leads to the reduction of energy storage costs, in terms of $ kW h^–1^ .^21−23^

The most, widely explored, chemical strategy to mitigate the voltage decay and structural degradation in LRLO is the optimization of the transition-metal blend.^4,5^ Incorporation of redox inactive metals, such as Al, Zr, and Ti, has been proposed in order to stabilize the lattice^24−26^ as well as the partial replacement of lithium ions with other alkali cations, for example, K and Na,^27^ or doping on the oxygen anion sublattice.^28^ As an example, Nayak et al.^24^ demonstrated that Al has both bulk and surface effects which improve the cyclability and reduce the drawbacks of LRLOs. Al doping on LRLOs results in a decrease of specific capacity but increases the stability upon cycling.

In this work, we demonstrate experimentally a strategy to extend the concept of LRLO to unexplored stoichiometries, landing to an optimized sample with stoichiometry Li_1.28_Mn_0.54_Ni_0.13_Co_0.02_Al_0.03_O_2−δ_. Our general strategy combines the simultaneous replacement of Co^3+^ centers in the layered structure with balanced amounts of Al^3+^ and Li^+^, exceeding for the last one, the common 1.2 stoichiometry coefficient, and inducing vacancies in the oxygen sublattice.

Generally speaking, we demonstrate a new class of over-lithiated materials, with a general formula Li_1.2+*x*_(Mn,Ni,Co,Al)_0.8–*x*_O_2−δ_, recently patented by us.^29^ This new class of materials combines the improved environmental benignity provided by the Co substitution with stabilized and enhanced electrochemical performance compared to stoichiometric nickel manganese cobalt or state-of-the-art LRLO.^30−32^ The complete series of the explored stoichiometries is reported in Supporting Information, Table S1. Among all the newly synthesized materials, we demonstrate the outstanding electrochemical properties in Li-ion batteries of the optimized sample with stoichiometry Li_1.28_Mn_0.54_Ni_0.13_Co_0.02_Al_0.03_O_2_ (OM) compared to the well-known LRLO benchmark Li_1.2_Mn_0.54_Ni_0.13_Co_0.13_O_2−δ_^30,33^ (BM).

## Experimental Methods

### Synthesis of the Over-Lithiated LRLO Li_1.2+*x*_Mn_0.54_Ni_0.13_Co_0.13–*x*–*y*_Al_*y*_O_2−δ_

The preparation of Li_1.2+*x*_Mn_0.54_Ni_0.13_Co_0.13–*x*–y_Al_y_O_2−δ_ was performed by the sol–gel method. Basically, stoichiometric amounts of lithium acetate [LiCH_3_COO·2H_2_O, Sigma-Aldrich], manganese acetate [Mn(CH_3_COO)_2_·4H_2_O, Sigma-Aldrich, 99.99% trace metals basis], nickel acetate [Ni(CH_3_COO)_2_·4H_2_O, Sigma-Aldrich, 99.995% trace metals basis], cobalt acetate [Co(CH_3_COO)_2_·4H_2_O, Alfa Aesar, 99.999% trace metals basis], and aluminum acetate [(HO)_2_Al(CH_3_COO), Sigma-Aldrich] were dissolved in ultrapure water. 5 wt % excess of lithium acetate was included in the synthesis to compensate the lithium loss during the high heating process. An aqueous solution of oxalic acid [C_2_H_2_O_4_ (Sigma-Aldrich)], acting as a chelating agent, was added to the metal-acetate solution to have a chelating agent/metal molar ratio of 1.5/1 and left under stirring. The pH in the mixture was maintained at 8 by the addition of ammonia solution [NH_4_OH, Sigma-Aldrich] dropwise. Then, the solution was dried slowly by heating at 80 °C and continuously stirred until a viscous mass was obtained. The as-obtained gel was finally completely dried at 200 °C under vacuum. This double-drying procedure was applied to control the particle morphology and size distribution as highlighted by Song and coworkers.^34^ To obtain the final product, two thermal treatments have been carried out in a muffle furnace in air. The resultant powder, finely milled, was preheated in the furnace at 450 °C for 2 h at an ambient atmosphere. The product was recovered from the furnace, milled using a mortar and pestle, and reheated 12 h at 900 °C at an ambient atmosphere.

### Material Characterization

Synchrotron diffraction patterns were collected at ELETTRA MCX beamline using a wavelength of 1.2 Å (10 keV) in the range between 10° ≤ 2θ ≤ 90°. Diffractograms were analyzed using Rietveld Refinement program GSAS-II.^35^ The elemental analysis was carried out by inductively coupled plasma optical emission spectrometry (ICP–OES), with an iCAP 7600 DUO Thermo Fisher Scientific. The morphology and composition of the materials were investigated by JEOL JSM-7500FA scanning electron microscopy (SEM), with a cold-field emission gun, equipped with an energy-dispersive X-ray (EDX) spectroscopy system based on an Oxford X-Max silicon-drift detector (80 mm^2^ active area). High-resolution transmission electron microscopy (HR-TEM) and high-angle annular dark-field scanning TEM imaging were carried out using JEOL JEM-2200FS TEM (Schottky emitter), operated at 200 kV, equipped with a CEOS corrector for the objective lens and an in-column image filter (Ω-type). Each sample powder was ground using an agate mortar and pestle and sonicated in toluene, and the supernatant was collected and drop-cast onto a holey-carbon-coated Cu grid. X-ray absorption spectra at the Mn and Ni K-edges were measured in the transmission mode at the X-ray absorption fine-structure (XAFS) beamline of the ELETTRA synchrotron radiation facility. A Si(111) double-crystal monochromator with an energy resolution of 0.8 eV at 7 keV was used. The intensity of the monochromatic X-ray beam was measured using three consecutive ionization chambers (Oxford) filled with suitable gas mixtures. Pelletized samples using polyethylene as a dispersing agent were placed in an evacuated sample chamber: the homogeneity of pellets has been checked before running the experiment. Reference spectra on Mn_2_O_3_ (Sigma-Aldrich, 99%), MnO_2_ (Sigma-Aldrich, 99%), NiO (Sigma-Aldrich, 99%), and LiNiO_2_ [synthesized from a stoichiometric mixture of Li_2_O and NiO at 700 °C for 12 h in air and checked by X-ray diffraction (XRD)] have been recorded as well under the same experimental conditions. X-ray absorption near-edge spectroscopy (XANES) spectra have been analyzed and fitted using Athena XAS data processing software. X-ray absorption spectra at the Mn, Ni, and Co L-edges have been measured at the advanced photoelectric effect high-energy (APE-HE) beamline at the ELETTRA synchrotron radiation source. The XANES spectra have been acquired using the total electron yield detection mode, allowing a probing depth of ∼5 nm. The energy resolution of these spectra is about 0.1 eV. The OM and BM samples, in the form of powders, have been glued on the sample holder using conductive silver paste and then loaded on the manipulator of the APE-HE chamber (under UHV conditions). The sample was oriented at 45° with respect to the incident beam, probing an area of ∼150 μm^2^. X-ray photoemission spectra of OM and BM were recorded exploiting a conventional nonmonochromatized X-ray source (Mg Kα = 1254 eV) with a hemispherical electron energy analyzer in a dedicated chamber of the NFFA UHV MBE-cluster system. Also, in this case, the powders have been glued on the sample holder using a conductive silver paste. The samples have been positioned at 45° with respect to the incident beam, probing an area of ∼1 mm^2^ and a depth of ∼1 nm. The Mn 2p spectra have been acquired using a pass energy of 50 and a dwell time of 2000 ms; they have been aligned using the Au VB spectra of a reference Au foil positioned just above the sample. Raman spectroscopy was carried out using a Dilor Labram instrument equipped with a He–Ne laser source at 632.7 nm and a CCD cooled detector. Si was used as the calibrating standard for the energy scale.

### Electrochemical Characterization

The positive electrode films were prepared from the active material, a conductive Super P carbon and polyvinylidene fluoride as a binder, in a weight ratio of 80:10:10. The powders are initially mixed, and then, 1-methyl-2-pyrrolidinone is added dropwise to form a slurry. The obtained slurry is casted onto an aluminum current collector using a doctor blade and cut into electrodes of 10 mm diameter. Finally, the electrodes were dried at 110 °C overnight. The as-prepared electrodes were assembled in Coin cells 2032 in an argon-filled dry glovebox, facing the as-prepared electrodes with metallic lithium disks (Sigma-Aldrich). A Whatman GF/D embedded with the LP30 electrolyte [1 M lithium hexafluorophosphate in ethylene carbonate/dimethyl carbonate (1:1 vol %), Solvionic] is used as the separator of the two electrodes. A Biologic BCS-805 battery cycler has been used for electrochemical tests. A charge/discharge activation procedure of cathode materials has been done, consisting of two cycles at 37.7 mA g^–1^, two cycles at 75.4 mA g^–1^, and two cycles at 377 mA g^–1^. Electrochemical tests of both materials have been carried out at 37.7 mA g^–1^ in the voltage range of 2–4.8 V (C-rate = C/5). Rate capability tests were performed by changing the current density from 37.7 up to 754 mA g^–1^ and then returning to 37.7 mA g^–1^.

Cyclic voltammetries at different scan rates have been performed on lithium cells with the use of a Biologic VMP-3 potentiostat.

Finally, complete lithium-ion cells were assembled using graphite (PI-KEM) as the negative electrode and tested using a MACCOR S4300 system in a voltage range from 2.2 to 4.7 V using a current density of 230 mA g^–1^for 300 cycles. The balance between the anode and cathode has been maintained considering the full initial capacity of graphite, and the cathode/anode mass ratio has been set at 1.75.

## Results and Discussion

### Over-Lithiated Li_1.28_Mn_0.54_Ni_0.13_Co_0.02_Al_0.03_O_2−δ_ (OM)

Li_1.28_Mn_0.54_Ni_0.13_Co_0.02_Al_0.03_O_2−δ_ (OM) is an over-lithiated Li-rich layered metal oxide, in which the content of cobalt has been highly reduced by codoping with lithium and aluminum. One cannot exclude the possible occurrence of oxygen vacancies in the final lattice (represented by δ in the stoichiometric formula), due to electroneutrality constraints, while replacing Co^3+^ ions in the BM with Li^+^ and Al^3+^ ions (see below for more details).

Following our approach, a series of materials have been synthesized starting from the parent Li_1.2_Mn_0.54_Ni_0.13_Co_0.13_O_2_ (BM) material to identify the optimal Li/Al/Co ratio and landing to the proposed optimized stoichiometry. All compositions have been checked by ICP–OES (see Supporting Information, Table S2) confirming the expected stoichiometries. The complete list of samples is presented in the Supporting Information (Table S1) where the physical–chemical characterization of all the over-lithiated materials is also discussed in the application note 1.

**Figure 1:**
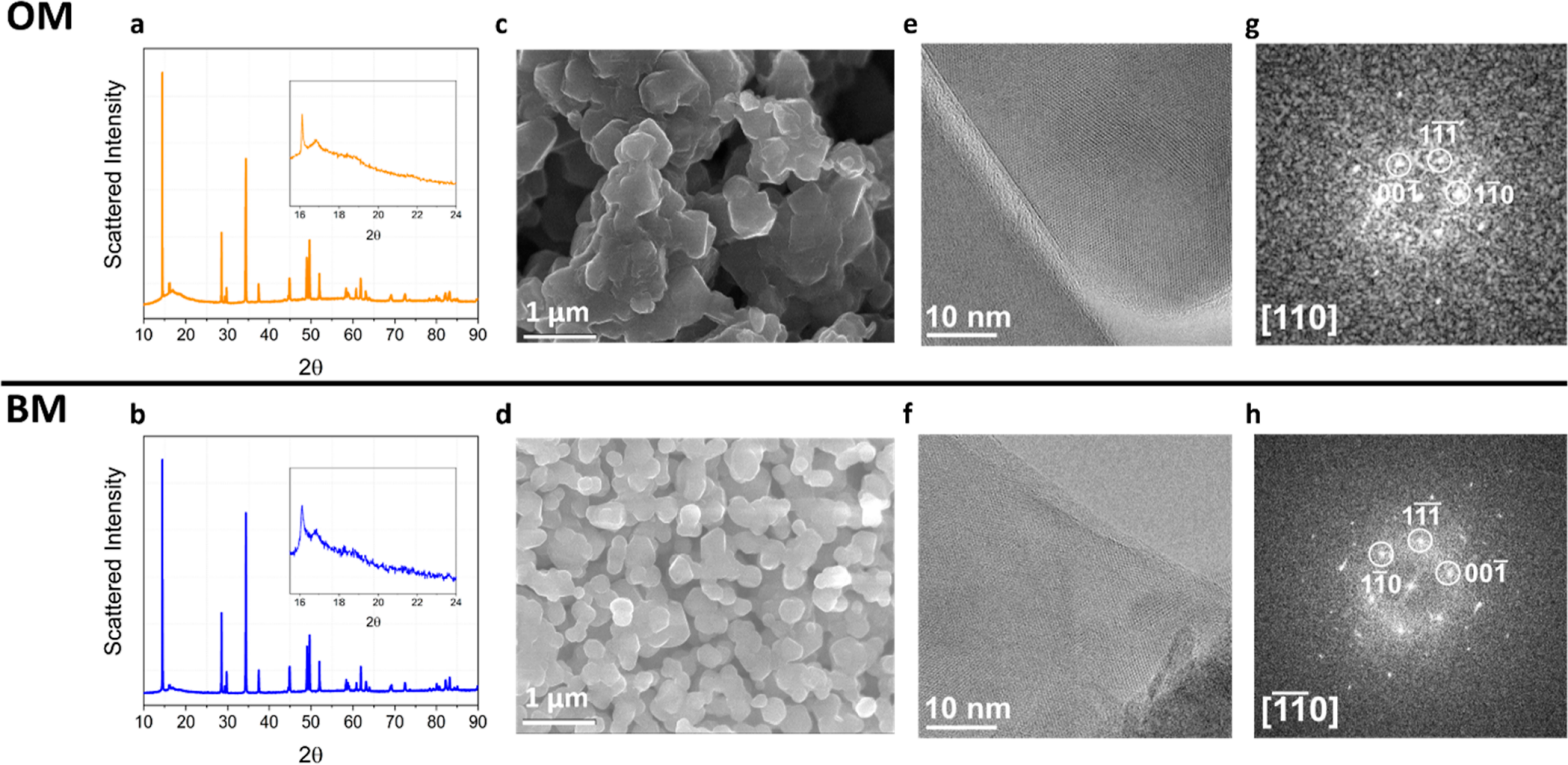
Structure and morphology of doped and pristine materials. (a,b) Synchrotron diffraction patterns, (c,d) SEM images, (e,f) HRTEM images of selected fragments, suspended on holes in the carbon film, and the (g,h) corresponding FFT patterns of samples OM and BM, respectively. The FFTs are indexed based on the mC24 structure (ICSD 202639).

Focusing on the optimized material, the structural and morphological comparison of OM and BM is presented in Figure 1 where synchrotron radiation XRD patterns, SEM, and HRTEM micrographs, as well as fast-Fourier transform (FFT)-reconstructed electron diffraction patterns, are shown.

From the morphological point of view, both BM and OM consist of agglomerates of submicron particles being the size of the OM particles around 400 nm, slightly larger compared to that of sample BM (see also Figure S4 in the Supporting Information). EDX spectroscopy maps shown in Figures S5 and S6 (Supporting Information) confirm that all elements are uniformly distributed without the evidence of phase segregation at the microscale.

Turning to the structure, synchrotron XRD patterns are similar for both BM and OM and prove the formation of a layered phase with coexisting hR12/mC24 structures. In fact, for both BM and OM, the diffraction patterns can be indexed by an hR12 lattice apart few extra peaks between 2θ of 16–25° (inset in Figure 1a,b). These weak and broad extra peaks are clues of the underlying monoclinic unit cell (mC24 lattice).^35−37^ Also, the HRTEM analysis confirms the same monoclinic local crystal structures of the samples OM and BM (see Figure 1e–h): for both samples, the electron diffraction patterns reconstructed by FFTs are indexed by the mC24 structure. Thus, cobalt substitution, aluminum doping, and over-lithiation do not alter the complexity of the structural identity of this material, where disordered mC24 unit cells almost randomly packed along the *c*-axis distort the long-range symmetry to a hR12 lattice. A discussion of the structural features of this complex lattice is summarized in the application note 2 in the Supporting Information.

Deeper insights about the structure of OM can be drawn by Rietveld refinements of synchrotron diffraction patterns (see the application note 2, Figures S7a,b and Tables S3–S4 in the Supporting Information). As expected, satisfactory refinement convergences can be obtained only assuming the hR12 structure, reaching the refinement assuming the mC24 one values of w*R* (%) above 9.6%.

Overall, the structure of OM compared to that of BM is almost unchanged, being the trigonal unit cell only marginally expanded (+0.1%) compared to BM: this small increase originates by a slight expansion of the lattice along the *c*-axis. The atomic occupancy disorder (antisite defect concentration) is also unaltered from BM to OM, whereas the oxygen occupancy decreases below unity in the OM material. This last change matches the charge unbalance originated by the replacement of Co^3+^ with Li^+^ and Al^3+^ (pseudo n-doping), thus confirming extended oxygen vacancies (i.e., estimated OM stoichiometry from XRD: Li_1.28_M_0.72_O_2−δ_, δ = 0.06, where M is the metal blend in the transition-metal layer).

The occurrence of oxygen vacancies induced by the pseudo n-doping impacts on the electronic structure of the OM compared to that of the BM as outlined by XANES at the K and L_2,3_-edges for Mn, Ni, and Co and X-ray photoemission spectroscopy in the Mn 2p region (see application note 3 and Figures S8–S10). L- and K-edges show that the surface composition is similar to the bulk one apart from minor electronic disorder from both materials. Quantitative analysis of the Ni and Mn K-pre-edges demonstrated the occurrence of a minor Jahn–Teller (JT) electronic disorder originated by the simultaneous charge transfer from the Ni^2+^ center to Mn^4+^ in both BM and OM. The formation of JT distortions leads to the formation of a small amount of Ni^3+^ and Mn^3+^ (∼6 and ∼11% of the Ni centers and ∼5 and ∼14% of the Mn ions for the BM and OM samples, respectively). One may notice that overlithiation and Co substitution in the OM material led to an increase of the JT defects compared to those of the BM one and to an increase in the net oxidation state of the nickel centers from +2.12 to +2.23 to mitigate the n-doping.

Starting from the ICP compositions and the XAS experimental charges on Ni and Mn in both OM and BM, it is possible to estimate the concentration of the oxygen vacancies, assuming the electroneutrality constraints and the Co^3+^ Al^3+^ Li^+^ oxidation states. Our estimates confirm the presence of vacancies on the oxygen anion sublattices only in the OM sample [i.e., δ = 0.1 assuming the general stoichiometry Li_1.28_TM_0.72_O_2−δ_ (OM)]. This defect concentration is in quantitative agreement with the XRD Rietveld results.

Once established, the impact of the alteration of the metal blend induced by overlithiation, that is, formation of extended oxygen vacancies and increase of JT distortions by minor oxidation of the Ni^2+^to Ni^3+^, it is possible to reconsider the apparent negligible structural changes of the lattice passing from BM to OM. By assuming the Shannon atomic radii for O^2–^ (1.40 Å), Mn^3+^ (0.58 Å), Mn^4+^ (0.53 Å), Ni^2+^ (0.69 Å), Ni^3+^ (0.56 Å), Co^3+^ (0.55 Å), Li^+^ (0.76 Å), and Al^3+^ (0.54 Å)^38,39^ and considering the volume of the unit cell derived by Rietveld refinement, it is possible to estimate the corresponding fraction filled by atoms in the transition-metal layer (2.8 and 3.0% for the BM and OM materials, respectively) as well as lithium ions in the Li layer (5.5% for both materials), oxygen anions in the O layers (68.7 and 66.5% for the BM and OM materials, respectively), or unoccupied (structural voids, 23.1 and 25.0% for the BM and OM materials, respectively). Overall, the minor structural change (+0.1% in the cell volume passing from BM to OM) balances opposite structural alterations: (i) the larger steric hindrance of the transition-metal layer and the formation of oxygen vacancies that (ii) shrinks the volume occupied by the anion sublattice and (iii) enlarge the empty voids in the lattice.

### Electrochemical Performance in Lithium Half-Cells

**Figure 2:**
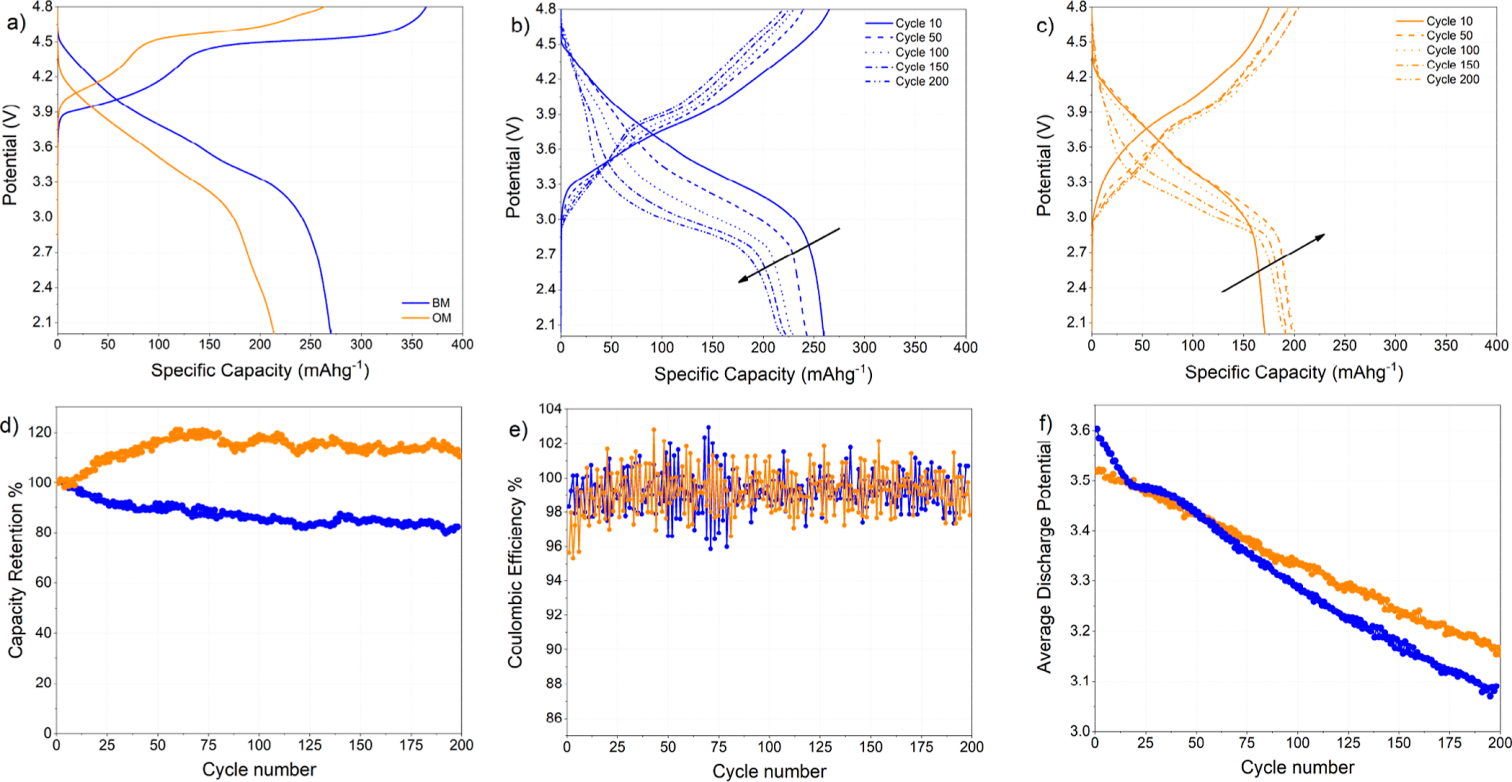
Comparison of electrochemical performance in lithium half-cells of OM and BM. Cells have been assembled according to the following galvanic chain: (−) Li/EC/DMC 1:1 vol LiPF_6_ 1 mol/L/LRLO (+) and tested in galvanostatic regimes at C/10 (37.7 mA g^–1^) in the 4.8–2.0 V vs Li range. (a) Potential profile of the first cycle of the galvanostatic cycling at C/10; (b) evolution of the potential profiles of the BM upon cycling; (c) evolution of the potential profiles of the OM upon cycling; (d) evolution of the capacity retention in discharge upon cycling calculated by dividing the discharge capacity at cycle *N* by the capacity recoded in the first electrochemical discharge of the cell; (e) comparison of the Coulombic efficiencies in lithium half-cells of BM and OM electrodes; and (f) comparison of the mean discharge potential upon cycling for the BM and OM electrodes.

The electrochemical performance of OM has been evaluated by galvanostatic charge/discharge cycling in lithium half-cells in comparison to that of BM, as shown in Figure 2.

Figure 2a reports the first charge/discharge profile obtained in the galvanostatic mode at C/10 between 2 and 4.8 V for the two samples. On passing, we underline that the used electrolyte, that is, LiPF_6_ 1 M in EC/DMC, is electrochemically stable in this voltage range, being the onset of the carbonate oxidation at 4.9–5 V versus Li.^40^ Upon charging, it is possible to distinguish two electrochemical processes: a first slope around 3.8 V, followed by a long plateau at 4.5 V. The first slope accounts for the oxidation of Co^3+^/Co^4+^ and Ni^2+^/Ni^4+^; while the process above 3.8 V is due to the oxidation of oxygen ions to peroxide.^41^ It is important to recall that along the 4.5 V plateau in the first charge, the oxygen peroxides partially disproportionate to release molecular oxygen, thus forming vacancies.^42,43^ Compared to the benchmark, OM exhibits relevant differences. Overall, OM is still able to exchange 264 mA h g^–1^ at the end of first charge to be compared to 363 mA h g^–1^ for BM. Furthermore, the first electrochemical oxidation of the OM occurs at slightly higher potentials than that of BM (around 4 V) and a remarkable shortening to the plateau at 4.5 V is observed. This phenomenon is an expected consequence of the removal of the redox active cobalt ions with redox inactive Li^+^ and Al^3+^.

Going beyond the first charge, the differences in the potential profiles between BM and OM are minor. In fact, in the first discharge and in all the following cycles, the potential curves proceed through a long slope in line with similar LRLO materials.^5,10,12^ The first discharge specific capacities are 271 and 214 mA h g^–1^, respectively, for BM and OM. The reduction in the discharge capacity recorded for the OM compared to that for the BM is counterbalanced by the remarkable improvement in Coulombic efficiency of the first cycle (i.e., 75% for BM and 81% for OM). Furthermore, the OM shows, as reported in Figure 2b,c, increasing discharge capacities upon cycling, whereas the BM material undergoes to the expected fading trend.^5,10,12^

The discharge capacity retention plot in Figure 2d confirms the superior cell performance of the OM compared to that of the BM for 200 cycles at the nominal current rate C/10 (corresponding to an effective rate of ∼C/5). In fact, the OM keeps an almost constant specific capacity value of about 200 mA h g^–1^ from cycle 50 to cycle 200, while the BM fades from 250 to 219 mA h g^–1^. With respect to the first charge capacity, the OM material shows an outstanding +44% increase in the capacity retention at cycle 200 with respect to BM. Further improvements can be highlighted in the cell performance of the OM compared to that of the BM: (a) an increase of the Coulombic efficiency (Figure 2e); (b) an increase of the mean discharge potential (Figure 2f) at cycle 200 is 3.16 V to be compared to 3.1 V vs Li for the BM material; (c) remarkable reduction of the potential hysteresis between charge and discharge (Figure 2f), that is, 0.7 V at cycle 200 to be compared to 0.93 V for BM; and (d) massive reduction of the cumulative irreversible capacity suffered upon cycling that is reduced by −38% at cycle 200 compared to BM (see Supporting Information, Figure S11).

Overall, the OM, despite the slight reduction in the specific capacity compared to the BM (−5% at cycle 200), shows superior capacity retention, smaller irreversible capacity, and improved energetic efficiency (i.e., 82 vs 76% at cycle 200 for Om and BM, respectively).

The CVs at different scan rates can be used to estimate the lithium diffusion coefficient in the Li insertion process, according to the Randles–Sevcik equation.^44^ The equation predicts the dependence of the value of the peak current on the square root of the scan rate to be linear. For both materials, Figure S12 shows the CVs at different scan rates and the corresponding linear fit of the peak current around 4 V (charge) against the square root of increasing scan rates. The *D*_Li_^+^ value of OM is half in comparison to that of BM, revealing only a slight decrease with respect to the large reduction of cobalt.

Turning to the rate performance (see Supporting Information, Figure S13a), the OM shows comparable power performance with respect to BM, despite the 6.5 times smaller cobalt content. The specific capacity of OM reaches around 120 mA h g^–1^ at a current of 754 mA g^–1^ and, furthermore, when the current decreases again to the initial value, capacity rises to the initial values of more than 200 mA h g^–1^. Moreover, OM can sustain high current better than BM; indeed, it maintains 58% of the initial capacity when the current reaches 754 mA g^–1^, while BM only maintains 55.7%. Moreover, we calculated the power density at different *C*_rate_ values (Figure S13b); in fact, the power density is another important parameter to take into account for the practical application. OM shows a comparable power density with respect to BM at low current density and a slight improvement at high current density. Despite the low content of cobalt, the power density is also very promising.

Among all beneficial effects in the cell performance provided by the substitution of Co with an optimized blend of Li/Al, the most relevant one is the strong mitigation of the voltage decay upon cycling shown in Figure 2b,c for the OM and BM, respectively. LRLOs suffer a remarkable voltage decay upon cycling due to the irreversible release of molecular oxygen, the accumulation of oxygen vacancies, and the resulting structural transformation from a layered to a spinel-like structure.^13,45−47^ The oxygen loss suffered during the first charge plateau at 4.5 V plays a crucial role in voltage fade^48^ as it impacts the redox active couple at high voltage O^2–^/O^–^ and the lattice stability.^49^ Therefore, the remarkable limitation of the voltage plateau in the OM compared to that in the BM is an electrochemical fingerprint of an improved balance between native vacancies and lattice stability, likely leading to the reduction of the voltage decay. To prove this point, the structural stability of OM has been evaluated postmortem XRD and Raman spectra of OM after 100 galvanostatic cycles at C/10, as shown in Supporting Information, Figure S14. The diffractogram, in comparison with the pristine one, highlights only a slight loss of crystallinity and cell volume expansion of the original hR12 lattice without any trace of extra peaks due to the spinel formation or other complex phase segregation (Figure S14a). Regarding the Raman analysis, the spectra of pristine LRLOs consist of two active modes connected to the hR12 lattice (600 and 485 cm^–1^) and six modes to the mC24 structure (605, 552, 432, 411, 364, and 328 cm^–1^).^49,50^ From the postmortem Raman spectra (Figure S14b), the most intense peaks around 600 and 485 cm^–1^ are still visible and intense for OM samples with a shoulder slightly shifted in the region of the spinel-like structure. On the other hand, in the BM sample, it possibly identifies only a broader peak around 620 cm^–1^. Instead, for both materials, the peaks associated with the mC24 structure disappear. The spectrum of OM confirms that the layered structure is still maintained. The results are in accordance with the postmortem XRD; the superstructure peaks are not even visible in the patterns and the layered structure is more defined in OM.

Finally, postmortem BM and OM samples have also been characterized by soft X-ray near-edge XAFS (NEXAFS) spectroscopy to evaluate changes in the valence of transition metals after the prolonged galvanostatic cycling test. In particular, Mn L_2,3_, Ni L_2,3_ and Co L_2,3_ edges have been acquired and the results are shown in Figure S15.

Looking at the upper panel of Figure S15a, one can observe a substantial modification of the chemical environment of the Mn sites, especially for the OM sample. In detail, after cycling, a new feature appears at ≈639 eV, labeled as structure A in Figure S15a. From the comparison with the reference spectra of MnO_2_, Mn_2_O_3_, and MnO, this new feature can be attributed to a Mn^2+^ oxidation state. Also, for what concerns the OM sample, the modifications are more pronounced, indeed the spectral shape is completely modified after cycling. From the comparison with the reference spectra, we also detected the presence of Mn^3+^ and Mn^4+^, indicating that different reduction processes occurred during the charge/discharge cycles.

Looking at the NiL_2,3_ spectra of Figure S15b, in this case, for the BM sample, no substantial spectral change has been detected with respect to the spectrum acquired on the pristine sample (Figure S10), indicating that a Ni^2+^ oxidation state is maintained in the postmortem sample. While in the case of the OM sample, some spectral modifications have been observed, in particular a decrease in the ratio between the intensities of structures E and F, parallel to the increase of the L_2_ edge features (located at ≈870 eV). From the comparison of the spectrum with the reference spectra in the bottom part of the figure, this spectral modification could be attributed to a Ni^2+^ → Ni^3+^ partial oxidation. Also, in this case, for OM, the modifications are more pronounced with respect to the BM sample.

Finally, Co L_2,3_ NEXAFS spectra of Figure S15c show one more time that the BM sample maintains its electronic structure after 100 cycles (Figure S10c), giving rise to the spectrum with a shape typical of Co^3+^. For the OM sample, a new feature appears at ≈777.5 eV (peak G), characteristic of a Co^2+^ oxidation state; this result indicates a Co^3+^ → Co^2+^ reduction consequent to the charge/discharge process.

### Demonstration in a Full Li-Ion Prototype

**Figure 3:**
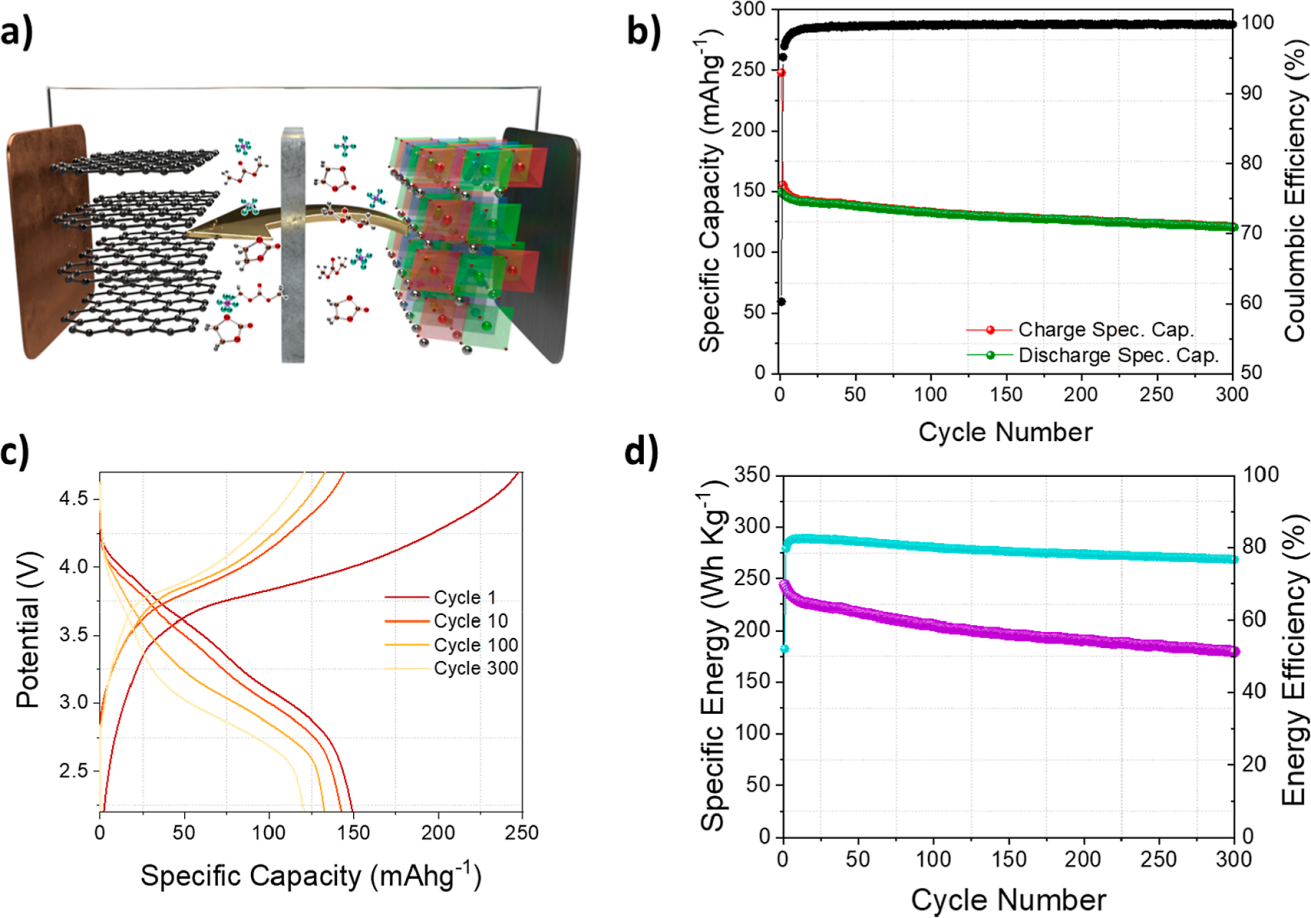
Full cell electrochemical test. Cells have been assembled according to the following galvanic chain: (−) graphite/EC/DMC 1:1 vol LiPF_6_ 1 mol/L/LRLO (+) and tested in galvanostatic regimes at 1 C (230 mA g^–1^) in the 4.7–2.2 V range. (a) Sketch of used full LIB. (b) Specific capacity vs cycle number plot; (c) voltage profile of selected cycles; and the (d) corresponding specific energy vs cycle. The specific capacity has been calculated with respect to the cathode mass.

Once established the superior performance of the optimized over-lithiated LRLO (OM) compared to the benchmark BM, we demonstrate its use as positive electrode in a full Li-ion formulation versus graphite to prove the performance of a full battery prototype. Battery performances are shown in Figure 3, whereas the electrochemical characterization of graphite lithium half-cells is reported in the Supporting Information (Figure S16). Before assembling the full battery, the electrode materials were activated in the lithium half-cell to stabilize the performances. The positive materials undergo the activation procedure described in the Experimental Methods section, while the negative material was cycled between 0.01 and 2 V.

The full LIB shows promising and stable performance upon cycling, reaching a specific discharge capacity of 150 mA h g^–1^, based on the mass of the positive material, and using a current density of 230 mA g^–1^ (1 C). The capacity retention after 300 cycles is 80.4% (Figure 3b). The voltage profile shows a featureless slope in charge and discharge with irreversible capacity losses limited to the first cycle being the Coulombic efficiency >99.5% from cycles 2–300. A remarkable constant specific energy is obtained reaching about 200 W h kg^–1^ at 1 C, calculated with respect to the sum of both electrode masses, with a stable energy efficiency approaching 80%. Additionally, the promising energy values and the energy retention are in line with previous results concerning LRLO materials, despite the low content of cobalt.^51−54^

## Conclusions

Herein, we propose and discuss a strategy to reduce the content of cobalt of a typical Li-rich material (i.e., Li_1.2_Mn_0.54_Ni_0.13_Co_0.13_O_2_) by codoping with lithium and aluminum, opening the door to overlithiation beyond the 1.2 stoichiometry paradigm. Through a rational balancing of aluminum and lithium content with respect to cobalt, we obtained an optimized material with the formula Li_1.28_Mn_0.54_Ni_0.13_Co_0.02_Al_0.03_O_2_. Using synchrotron radiation diffraction experiments, we proved that the layered structure of Li-rich materials is maintained after the replacement with aluminum and lithium. The small alteration of the lattice results from the balancing of concurrent effects, namely, a larger steric hindrance of the transition-metal layer and the formation of oxygen vacancies. Despite a slight decrease of the specific capacity, the performance of OM in lithium half-cells is remarkably improved, thanks to the reduction of the irreversible capacity accumulation, improvement of the capacity retention, increase of the mean discharge potential, and limitation of the potential hysteresis between charge and discharge. In particular, a remarkable improvement in the voltage decay is observed originated by the excellent structural stability of OM upon cycling. As a final point, the implementation of OM in a full LIB has been demonstrated with excellent performance at high current rates.

## Supporting Information Available

Additional experimental details including XRD pattern refinement, ICP–OES results, SEM–EDS micrographs, X-ray spectroscopies, rate capability test, and postmortem analysis (PDF)
