# Supplementary material for: Pushing Stoichiometries of Lithium-Rich Layered Oxides Beyond Their Limits
Source: ACS Appl Energy Mater. 2022 Feb 11;5(2):1905–13. doi: 10.1021/acsaem.1c03396 (PMC8889532; doi:10.1021/acsaem.1c03396)
Supplement: Supplementary file 1 — ae1c03396_si_001.pdf [file ae1c03396_si_001.pdf]

Supporting Information

Pushing stoichiometries of Lithium Rich Layered Oxides beyond their limits

Arcangelo Celeste,[1,2] *Rosaria Brescia,*[3] *Giorgia Greco,*[4] *Piero Torelli*[5]*, Silvia Mauri*[5,6]*, Laura Silvestri,*[7] *Vittorio Pellegrini*[2,8]*, Sergio Brutti,*[4,9,10,*]

[1]Dipartimento di Chimica e Chimica Industriale, Università degli Studi di Genova, via Dodecaneso 31, 16146 Genova, Italy

[2]Graphene labs, Istituto Italiano di Tecnologia, via Morego 30, 16163 Genova, Italy

[3]Electron Microscopy Facility, Istituto Italiano di Tecnologia, via Morego 30, 16163 Genova, Italy

[4]Dipartimento di Chimica, Università di Roma La Sapienza, p.le Aldo Moro 5, 00185 Roma, Italy

[5]Istituto Officina dei Materiali (IOM)–CNR, Laboratorio TASC, Area Science Park, S.S.14, km 163.5, I-34149 Trieste, Italy

[6]Dipartimento di Fisica, University of Trieste, via A. Valerio 2, 34127, Trieste, Italy

7Dipartimento di Tecnologie Energetiche e Fonti Rinnovabili, ENEA C.R. Casaccia, via Anguillarese 301, 00123 Roma, Italy

[8]BeDimensional Spa, via Torrentesecca 3d, 16163 Genova, Italy

[9]GISEL—Centro di Riferimento Nazionale per i Sistemi di Accumulo Elettrochimico di Energia, INSTM via G. Giusti 50121 Firenze, Italy

[10]ISC-CNR OUS Sapienza, Via dei Tarquini, 00185 Roma, Italy

* corresponding author. Email sergio.brutti@uniroma1.it

Application note 1.

Structural and physico-chemical characterization of the entire series of over-lithiated materials.

The homologue series of samples synthesized from the BM by substitution of cobalt with lithium and aluminum beyond is listed in the table S1.

Table S1. Series of synthetized materials with different levels of Cobalt, Aluminum and Lithium.

Name	Chemical formula	Li%	Al%	Co%
**BM**	$Li_{1.2}Mn_{0.54}Ni_{0.13}Co_{0.13}O_2$	60	0	6.5
**S01**	$Li_{1.2}Mn_{0.54}Ni_{0.13}Co_{0.1}Al_{0.03}O_2$	60	1.5	5
**S02**	$Li_{1.2}Mn_{0.54}Ni_{0.13}Co_{0.07}Al_{0.06}O_2$	60	3	3.5
**S03**	$Li_{1.2}Mn_{0.54}Ni_{0.13}Co_{0.04}Al_{0.09}O_2$	60	4.5	2
**S04**	$Li_{1.23}Mn_{0.54}Ni_{0.13}Co_{0.07}Al_{0.03}O_2$	61.5	1.5	3.5
**S05**	$Li_{1.26}Mn_{0.54}Ni_{0.13}Co_{0.04}Al_{0.03}O_2$	63	1.5	2
**S06**	$Li_{1.26}Mn_{0.54}Ni_{0.13}Co_{0.02}Al_{0.05}O_2$	63	2.5	1
**OM**	$Li_{1.28}Mn_{0.54}Ni_{0.13}Co_{0.02}Al_{0.03}O_2$	64	1.5	1

Atomic composition has been checked by Inductively Coupled Plasma Optical Emission Spectroscopy (ICP-OES, see table S2) confirming the expected stoichiometries in all cases.

Table S2. ICP-OES results of samples.

Sample	Theoretical Ratio Ni:Co:Al:Mn	Experimental Ratio Ni:Co:Al:Mn
**BM**	0.241:0.241:0:1	0.272:0.284:0:1
**S01**	0.241:0.185:0.056:1	0.247:0.206:0.053:1
**S02**	0.241:0.13:0.111:1	0.253:0.14:0.092:1
**S03**	0.241:0.074:0.167:1	0.247:0.079:0.156:1
**S04**	0.241:0.13:0.056:1	0.25:0.145:0.053:1
**S05**	0.241:0.074:0.056:1	0.247:0.082:0.054:1
**S06**	0.241:0.037:0.093:1	0.246:0.042:0.089:1
**OM**	0.241:0.037:0.056:1	0.241:0.041:0.055:1

The X-ray diffraction (XRD) patterns of all samples have been recorded using a Malvern PANalytical Empyrean (Cu Kα) in the range of 2θ degree of 10°−90°, as shown in the figure S1. All diffraction patterns display a layered phase for all samples, indicating the absence of modification of the crystal structure due to the Li and Al co-doping. In fact, similarly to BM, the diffraction pattern can be indexed by adopting an hR12 lattice with R-3m symmetry typical of the $LiMO_2$ layered phases. The extra peaks observed between 2θ of 20°-30° are identified in line with

the literature with a coexisting mC24 lattice with C2/m symmetry generally reported as Li_2MnO_3 phase (see figure S2 for a representation of both trigonal and monoclinic unit cells).

The two lattices, i.e. hR12 and mC24, are closely related by symmetry: both structures are constituted by the layering of Li/O/M/O planar sheets (M=metal blend) in the (001) direction. The composition of the metal blend in the M-layer tunes the final stoichiometry that can easily range in both structures from $Li(Li_{1/3}TM_{2/3})O_2$ to $LiTMO_2$ (TM= transition metals). The main differences between the two lattices deals with the different stacking sequence as well as the ordering in the M layer, that is regularly constituted by $LiTM_6$ motifs in the mC24 phase whereas is completely random in the hR12 one. Overall, the two lattices can describe the same stoichiometry but can be easily discriminated by diffraction thank to the extra peaks and differences in the peak intensity (see the reference patterns in the figure S1). However, the occurrence of a partial break in the periodicity of the mC24 lattice (e.g. staking faults, atomic site occupation disorder) makes the effective structure very similar to the hR12, thus producing a diffraction pattern almost indistinguishable compared to the trigonal phase, apart the observed weak peaks (see above). [3–5]

All over-lithiated samples show similar morphologies as observed by scanning electron microscopy (see figure S3).

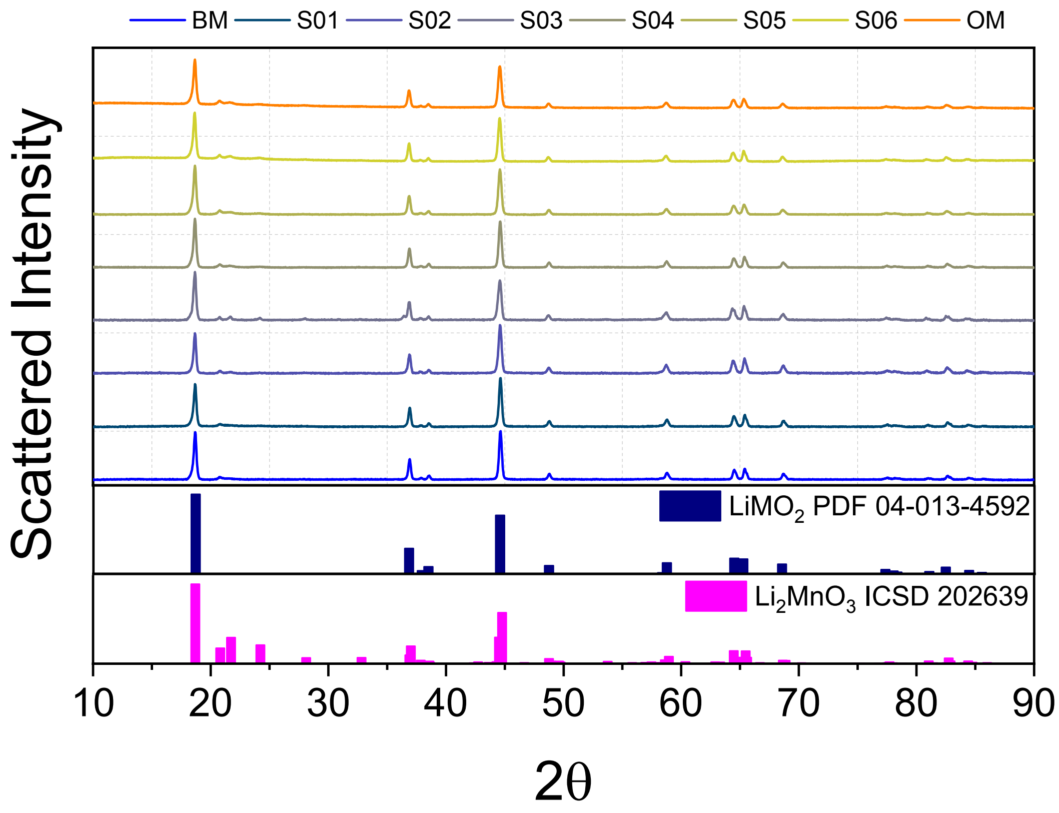

Figure S1. XRD patterns of all over-lithiated Li-Rich layered materials.

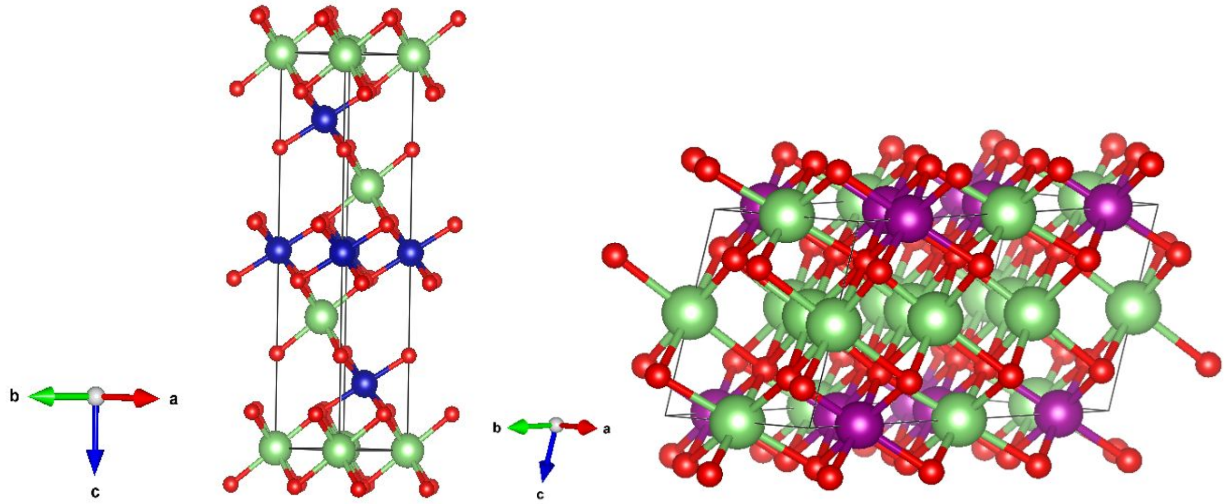

Figure S2. Representation of Hexagonal cell and Monoclinic cell.

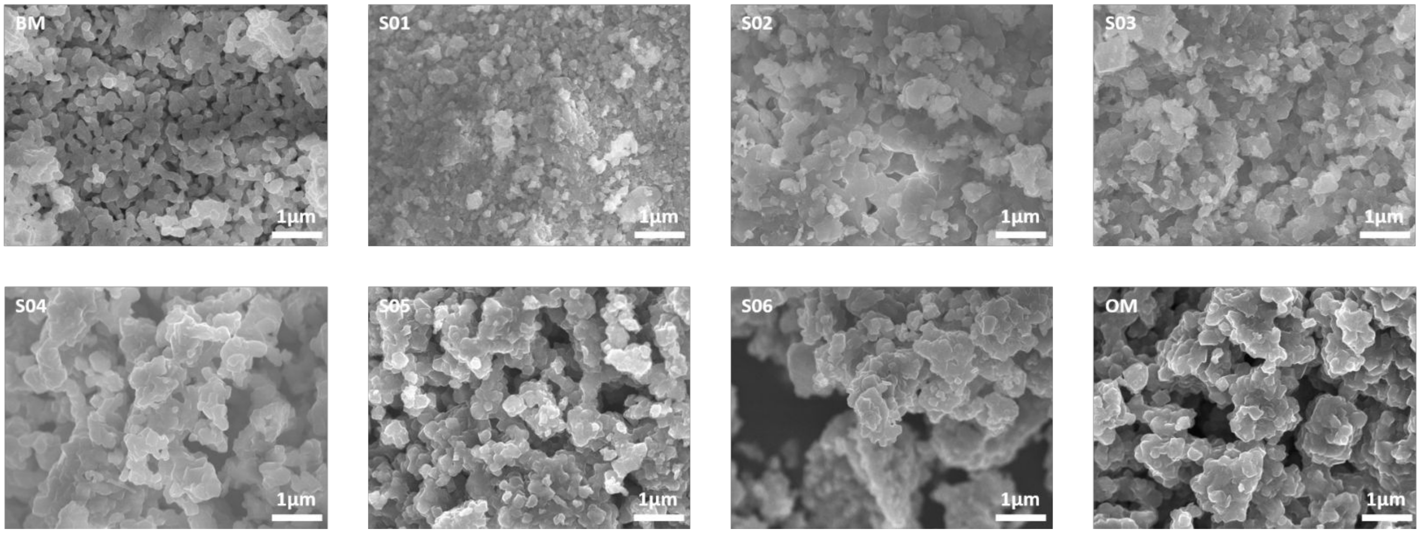

Figure S3. Comparison of the morphologies of the over-lithiated layered oxides by secondary electron-scanning electron microscopy imaging.

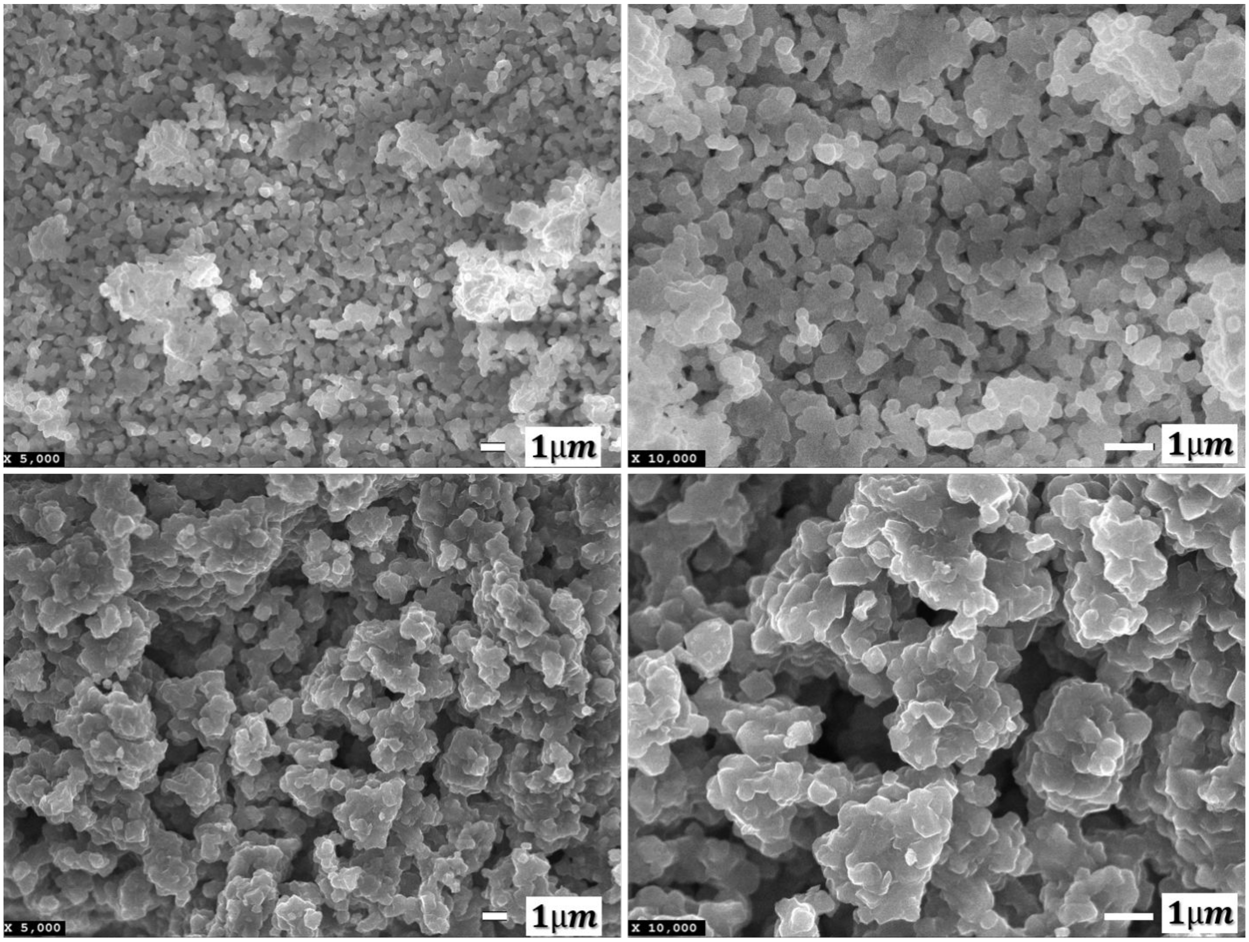

Figure S4. Secondary Electron-SEM imaging of OM (a, b) and BM (c, d) at different magnification.

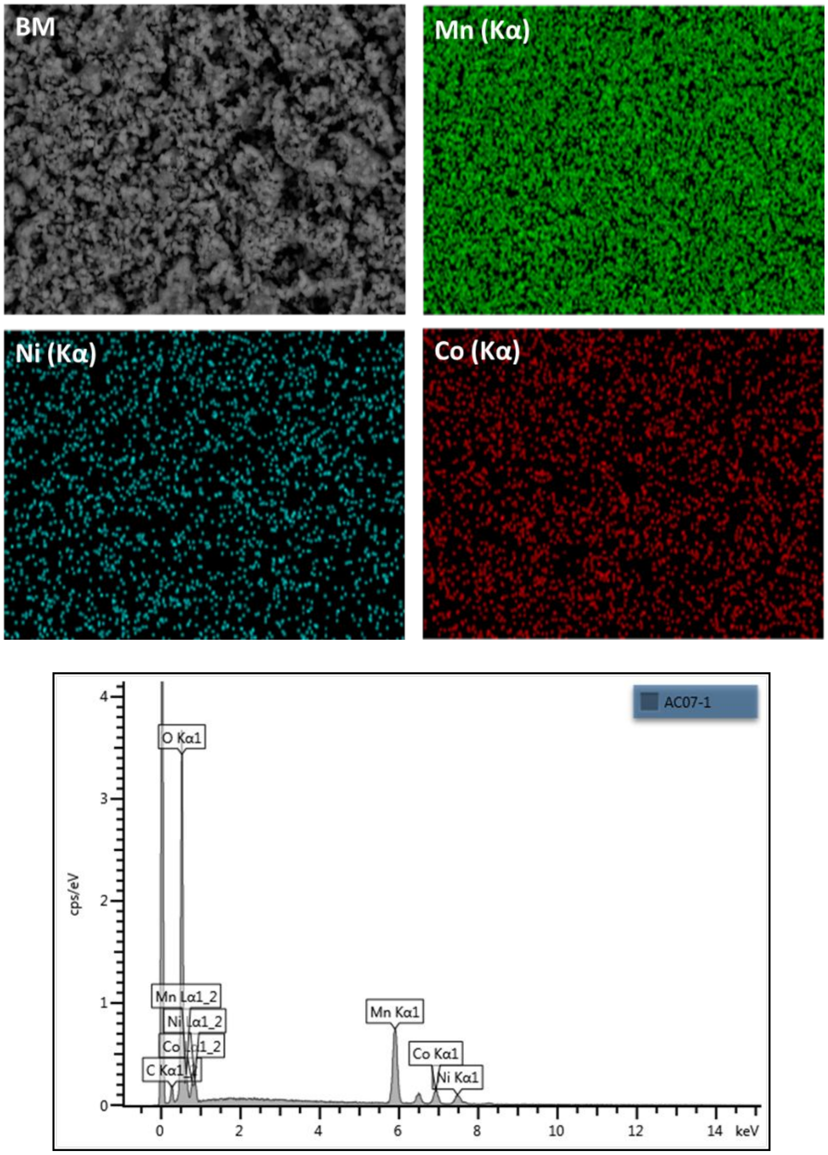

BM	Line Type	Atomic %
**O**	K series	66.92
**Mn**	K series	21.85
**Co**	K series	6.18
**Ni**	K series	5.05
**Total**		100.00

Figure S5. BM EDX maps.

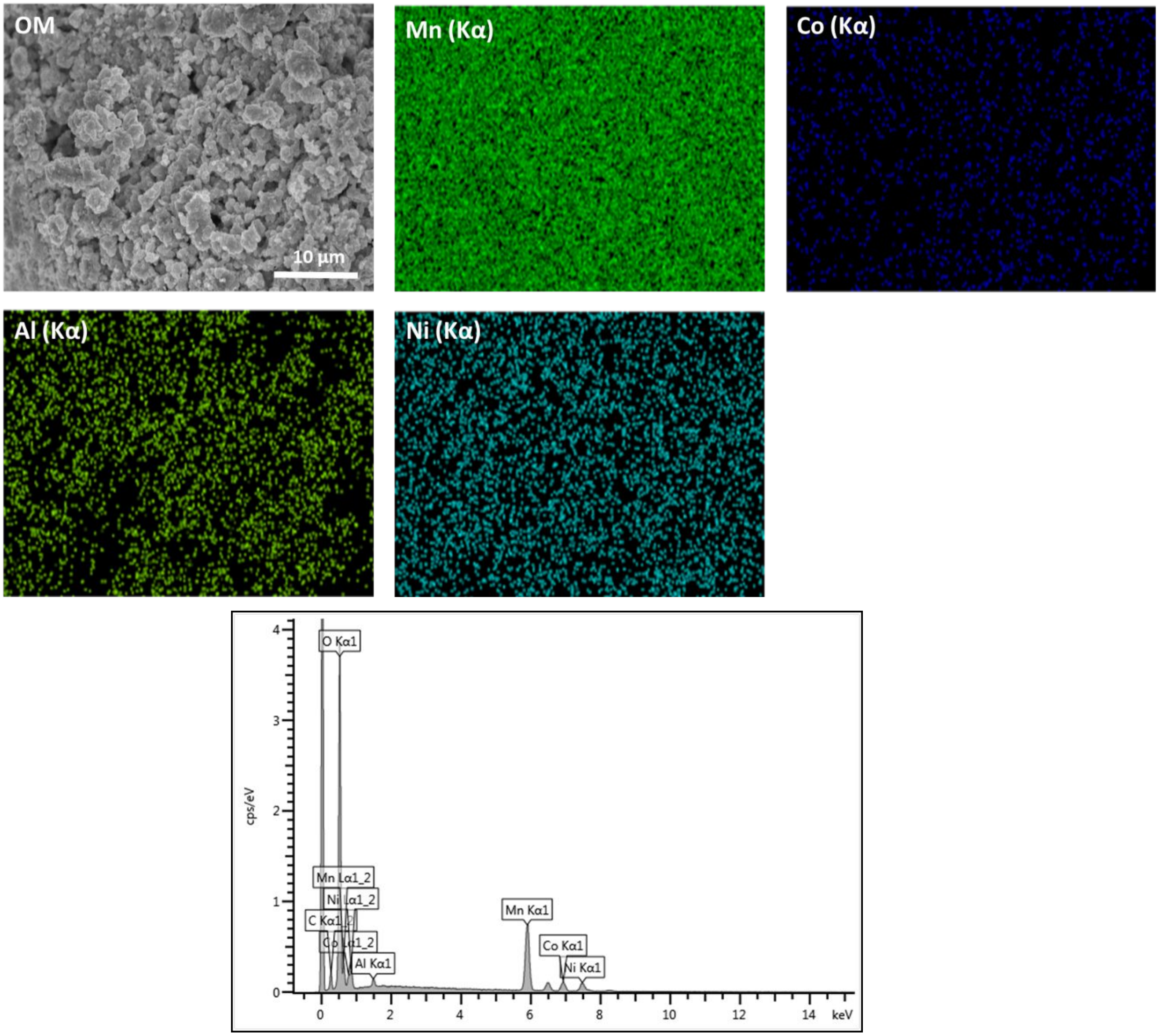

OM	Line Type	Atomic %
**O**	K series	68.42
**Al**	K series	1.09
**Mn**	K series	20.83

**Co**	K series	4.60
**Ni**	K series	5.06
**Total**		100.00

Figure S6. OM EDX maps.

Application note 2.

Rietveld refinements of the BM and OM materials

Synchrotron XRD patterns were analyzed by Rietveld Refinement program GSAS-II[1]. The hR12 and the mC24 structures have been both adopted starting from the prototypes, i.e. $LiCoO_2$ and Li_2MnO_3, respectively. [6,7] The prototypal crystal structures are summarized in the table S3: atomic occupancies have been updated to mimic a generic stoichiometry $Li_{1.2}(Co,Mn,Ni)_{0.8}O_2$.

Table S3. Prototypal lattices used for the Rietveld refinements[6,7]. Occupancy fractions are calculated for the generic stoichiometry $Li_{1.2}(Co,Mn,Ni)_{0.8}O_2$

	**mC24**	**hR12**
**Space group**	C 2/m	R-3m
**Lattice parameters**	a≠b≠c<br>α= γ =90˚ β=109.3˚	a=b≠c<br>α=β=90˚ γ=120˚
**Atomic position and occupancies (Wycoff position; OF=occupancy fraction)**		

**Lithium ion layer**	(2c) (0 0 ½)<br><br>OF(Li)=1	(3b) (0 0 ½)<br><br>OF=1
	(4h) (0 0.661 ½)<br><br>OF(Li)=1	
**Metal ions blend layer**	(2b) (0 ½ 0)<br><br>OF(Li)=0.6<br>OF(Mn+Co+Ni)=0.4	(3a) (0 0 0)<br><br>OF(Li)=0.2<br>OF(Mn+Co+Ni)=0.8
	(4g) (0 0.167 0)<br><br>OF(Mn+Co+Ni)=1	
**Oxygen ions layers**	(4i) (0.219 0 0.227)<br><br>OF(O)=1	(6c) (0 0 z)<br><br>OF=1
	(8j) (0.254 0.321 0.223)<br><br>OF(O)=1	

The following parameters have been optimized in all refinements: lattice parameters, atomic positions, crystallite size, lattice strain, atomic positions, Debye-Waller factors, oxygen occupancy fraction to mimic vacancies and Li/Ni antisite defect concentration. The antisite defect concentration has been evaluated by optimizing the fraction occupancies of Li and Ni in the (3b) and (3a) hR12 sites or in the (4h) and (4g) mC24 sites also applying a stoichiometry constraint to preserve the overall composition. The final wR(%) values for the BM refinements are 4.55% and

7.9% whereas for the OM refinements are 6.91% and 9.6% for the hR12 and mC24 lattices, respectively. These results confirm the reasonable assumption to represent the structural complexity of LRLO with an approximate hR12 lattice. The comparison between experimental and refined XRD patterns for both BM and OM materials are shown in the figure S7 whereas the optimized structural parameters are analytically reported in the table S4

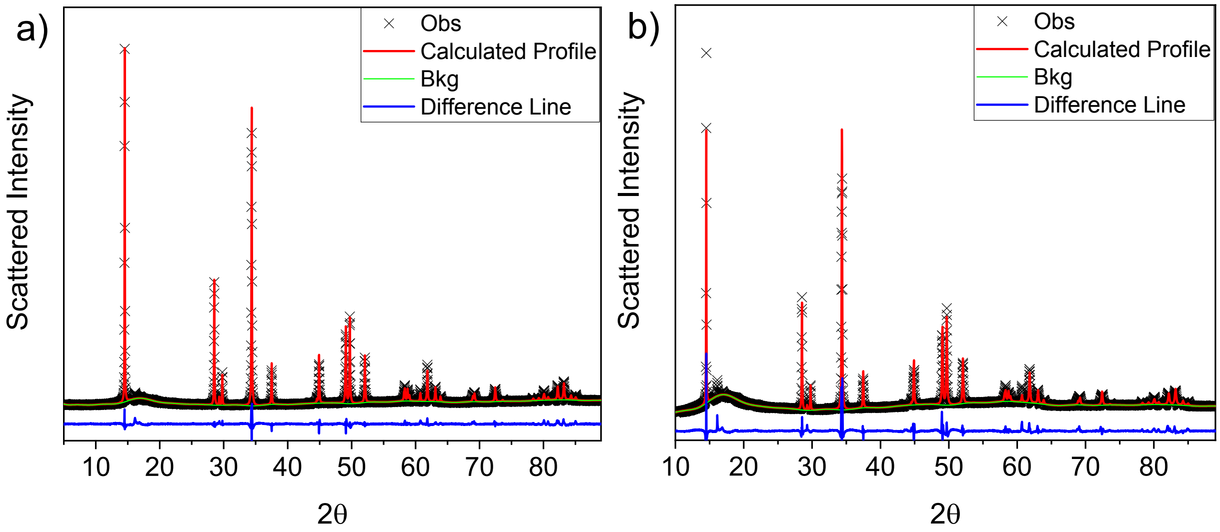

Figure S7. Experimental and Rietveld Analysis of BM (a) and OM (b). The calculated patterns are shown by red line, and the black cross show the observed intensities. The differences between the observed and calculated intensities are presented by blue curves.

Table S4. Refinement parameters of BM and OM.

**Stoichiometry**				**wR(%)**	
**$Li_{1.2}Mn_{0.54}Ni_{0.13}Co_{0.13}O_2$**				**4.55**	
Structural Model	*Cell Parameters*	*Atoms*	*Wyckoff Position*	*Atomic Coordinates*	*Occupancies*
R-3m	$a = 2.854$<br>$b = 14.239$	Li/Ni<br>Mn/Co/Ni/Li<br>O	3b<br>3a<br>6c	0, 0, 0.5<br>0, 0, 0<br>0, 0, 0.739	0.99/0.01<br>0.54/0.13/0.12/0.21<br>1.0
**Stoichiometry**				**wR(%)**	
**$Li_{1.28}Mn_{0.54}Ni_{0.13}Co_{0.02}Al_{0.03}O_2$**				**6.91**	
Structural Model	*Cell Parameters*	*Atoms*	*Wyckoff Position*	*Atomic Coordinates*	*Occupancies*
R-3m	$a = 2.855$<br>$b = 14.248$	Li/Ni<br>Mn/Co/Ni/Li/Al<br>O	3b<br>3a<br>6c	0, 0, 0.5<br>0, 0, 0<br>0, 0, 0.739	0.99/0.01<br>0.54/0.02/0.12/0.29/0.03<br>0.97

Application note 3.

X-ray spectroscopies on LRLO materials

The XANES spectra at the Ni and Mn K-edges are shown for the BM and OM materials in the figure S8.

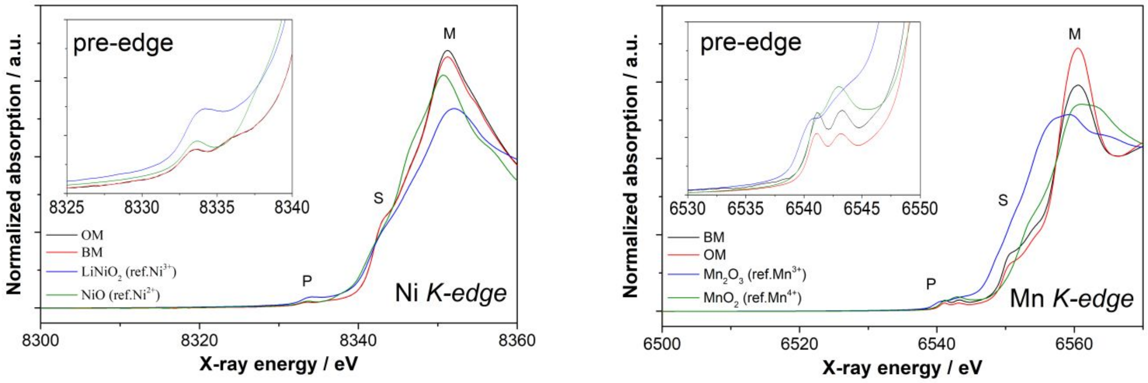

Figure S8. Experimental XANES spectra at the K-edges of Ni and Mn measured for the BM and OM samples. Experimental reference spectra for Mn_2O_3, MnO_2, NiO and $LiNiO_2$ are also shown.

The two samples show mainly the same electronic structure at both Mn and Ni edges. Pre-edge peak P (1s → 3d inner atomic transition, dipole forbidden- quadrupole allowed) at Mn edge suggests the occurrence of a reduced inversion symmetry of Jahn-Teller distorted MnO_6 octahedra in the structure, being the observed doublet due to the crystal-field splitting of Mn 3d states into

t_{2g} and e_g orbitals. The other spectra features (e.g. S peak associated with 1s transitions into p-like states of t_{1u} symmetry, and the white-line M)[8] are remarkably similar in the two samples. Quantitative fittings of the pre-edge and edge regions allow to estimate the mean Ni and Mn oxidations states in the two samples (i.e. Ni +2.12±0.02 and +2.23±0.02, Mn+3.89±0.02 and +3.86±0.02, for BM and OM, respectively). These values confirm the occurrence of a minor Jahn-Teller electronic disorder distortion originated by the simultaneous charge transfer from the Ni^{2+} centers to Mn^{4+}, thus leading to the formation of small amounts of Ni^{3+} and Mn^{3+}, as already seen in the L edges. A careful analysis of the oxides stoichiometry, assuming the electroneutrality constraints and the Co^{3+} Al^{3+} Li^{+} oxidation states, indirectly confirms the possible occurrence of vacancies on the oxygen anion sublattices in the Co-poor sample (i.e. z≈0 and 0.1, $Li_{1+x}M_{1-y}O_{2-z}$, for BM and OM, respectively) in line with the XRD Rietveld results.

The XANES spectra at the Co, Ni and Mn $L_{2,3}$-edges are shown in the figure S10.

Fig. S9 shows the $L_{2,3}$ XANES spectra of Mn, Ni and Co in OM and BM samples. The electronic transitions giving rise to the most intense structures of these spectra are related to dipole allowed $2p \rightarrow 3d$ transitions. The spin–orbit interaction of the 2p core holes splits the spectra into two multiplets, the L_3 ($2p_{3/2}$) edges at ≈ 643, 853 and 780 eV for respectively Mn, Ni and Co, while

the L_2 ($2p_{1/2}$) edges fall at ≈ 653, 871 and 795 eV. In turn, all the three L_3 are split in two structures (labeled as A and B). Since the $L_{2,3}$ edges probe the unoccupied 3d states of the absorbing atoms, the resulting spectral shapes change accordingly to the different filling of the 3d orbitals in the ground state (and thus to different oxidation numbers) but also to the different crystal field effects[9]. The comparison with the reference spectra (lower panels) allows to assign the oxidation numbers of 4+, 2+ and 3+ respectively to Mn, Ni and Co for both samples[10,11].

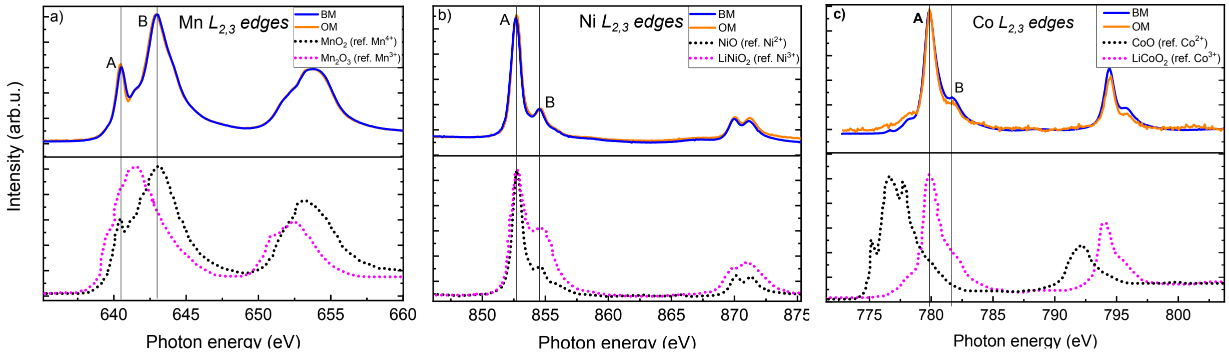

Figure S9. Upper panels: experimental L2,3 edges XANES of a) Mn, b) Ni, c) Co measured for the S01 (blue line) and S08(red line) samples. Lower panels: experimental reference spectra for a) Mn_2O_3 and MnO_2, b) NiO and $LiNiO_2$, c) $LiCoO_2$ and CoO.

Focusing on the Mn $L_{2,3}$ edges spectra, peaks A and B are related to $p \rightarrow d$ dipole allowed transitions towards the unoccupied 3d molecular orbitals originating from the crystal-field splitting

of Mn 3d states in a distorted octahedral geometry[10]. Comparing the experimental spectra with the reference spectrum of the MnO_2 (black dotted spectrum), it can be observed that A and B structures in the experimental spectra are sharper. This can be related to transitions towards more energetically localized 3d orbitals, confirming thus that in both OM and BM samples the orbital splitting caused by the Jahn Teller effect is less pronounced with respect to the conventional distorted octahedral coordination of Mn in MnO_6. It is important to underline that this last consideration is only qualitative, indeed the energy separation between peaks A and B, albeit depending on the crystal field effects, cannot be related directly to the Dq between the t_{2g} and e_g states, because the 3d-3d as well as the 2p-3d two-particle interactions strongly contribute to the spectral shape[9]. For what concern Ni^{2+} and Co^{3+}, also theoretical calculations confirm that structures A and B in the L_3 edges spectra are related to an O_h symmetry of the absorbing atoms[4].

The data of *L edge* show that the surface composition of the sample is similar to bulk one observed in the XAS *K edges* spectra of Fig. S9: indeed, the soft X-Rays spectroscopy (operated in total electron yield) is much more surface sensitive than the hard X-Rays one (related to the photon energies of the transition metals K edges).

The XPS spectra of the Mn 2p region has been also acquired and analyzed for both BM and OM to match the results of the XANES investigation (see figure S11). The Mn 2p core levels of OM and BM (see figure S10) confirms the +4 oxidation state of Mn (looking at the spectral shape and energetic positions[13]). The two peaks slightly shift (≈0.2 eV) to lower binding energies in OM possibly attributed to a minor oxidation of the Mn centers in agreement with the quantitative fitting of the K edge XANES spectra of Mn reported above.

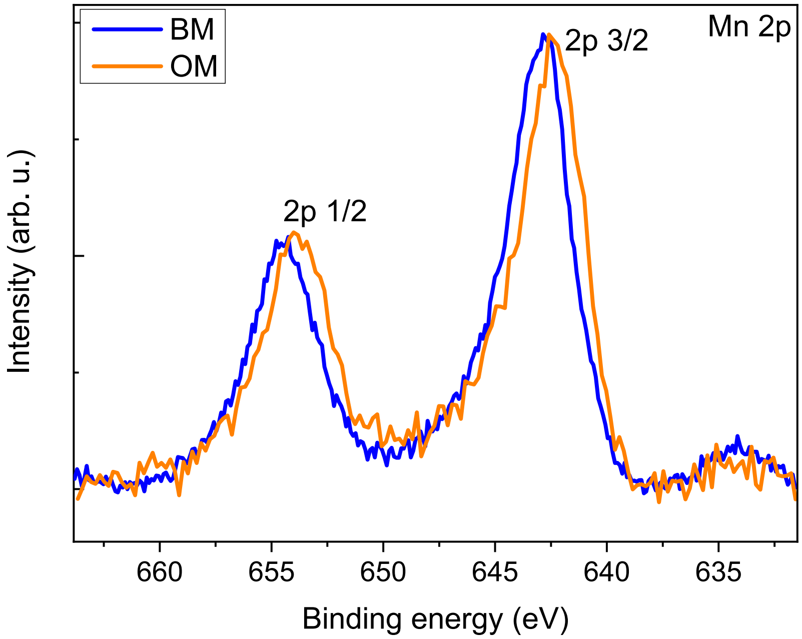

Figure S10. Experimental XPS Mn 2p core level spectra of S01 and S08 samples. 2p 3/2 ≈ 642.5 eV; 2p ½ ≈ 654 eV.

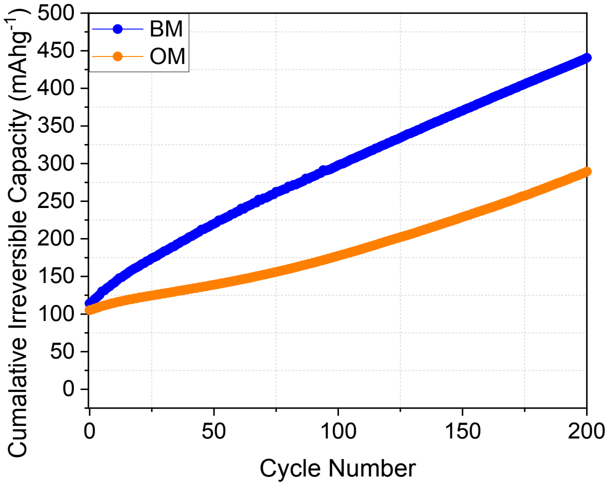

Figure S11. Cumulative irreversible capacity vs cycle number.

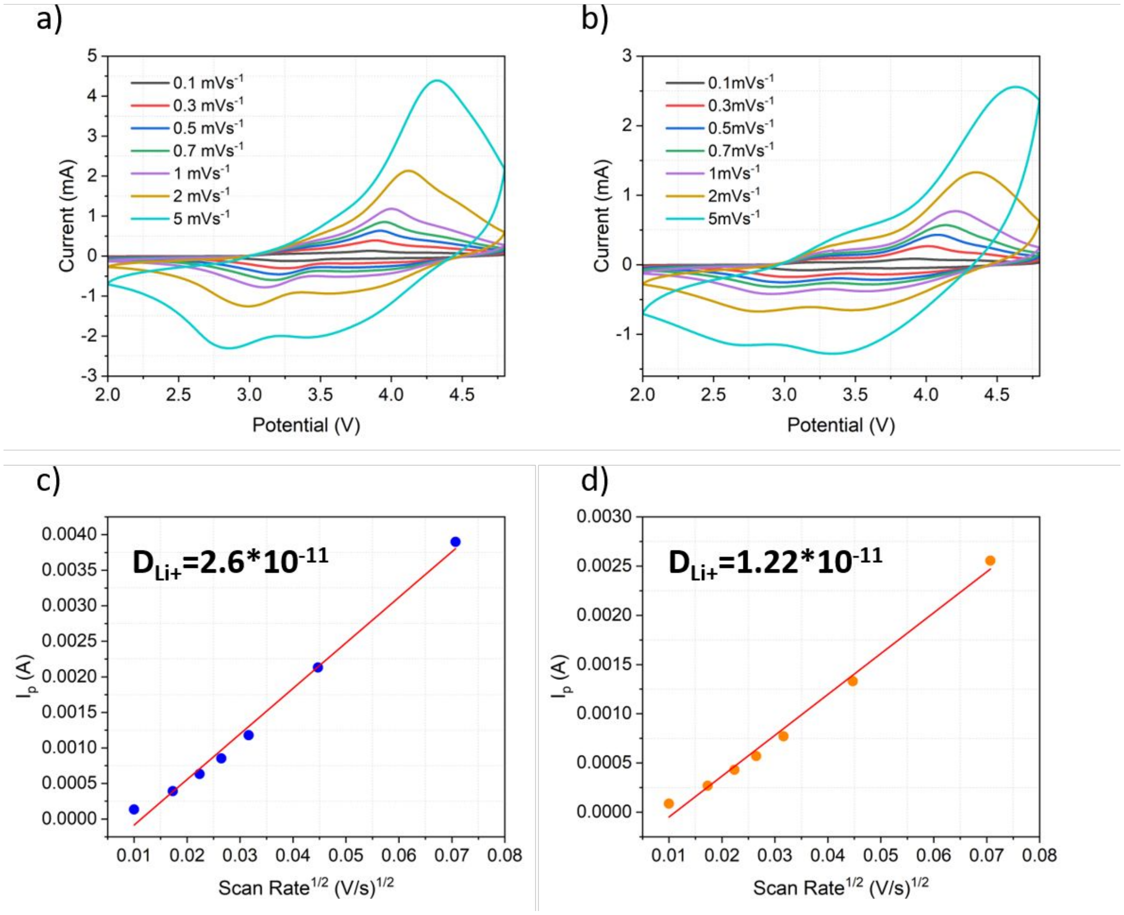

Figure S12. a) BM and b) OM cyclic voltammetry curves at increasing scan rates. c-d) Corresponding Randles–Sevcik analysis of the peak current density against the square root of scan rates.

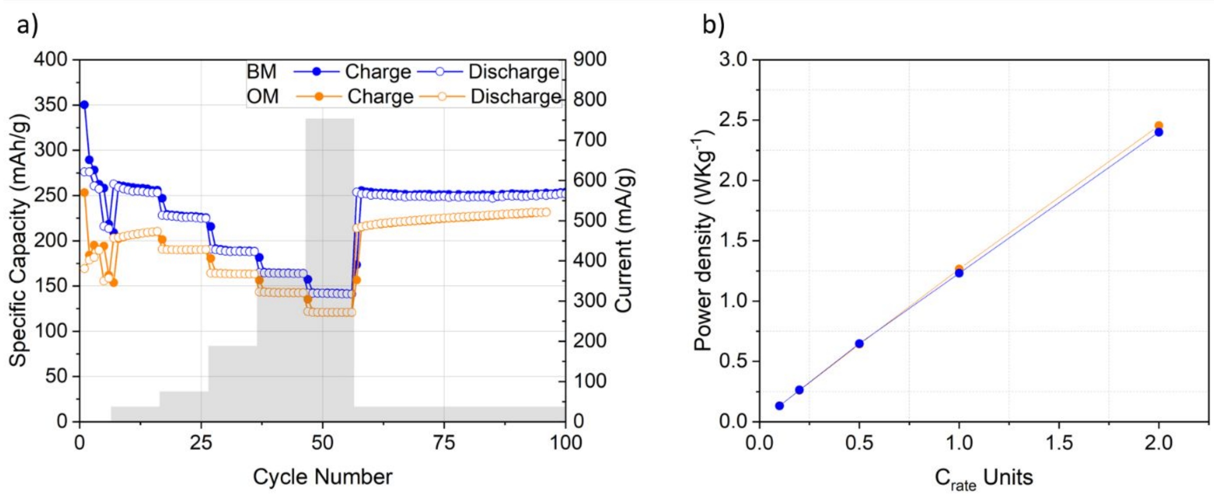

Figure S13. a)Rate capability test. b)Power density vs C_{rate}.

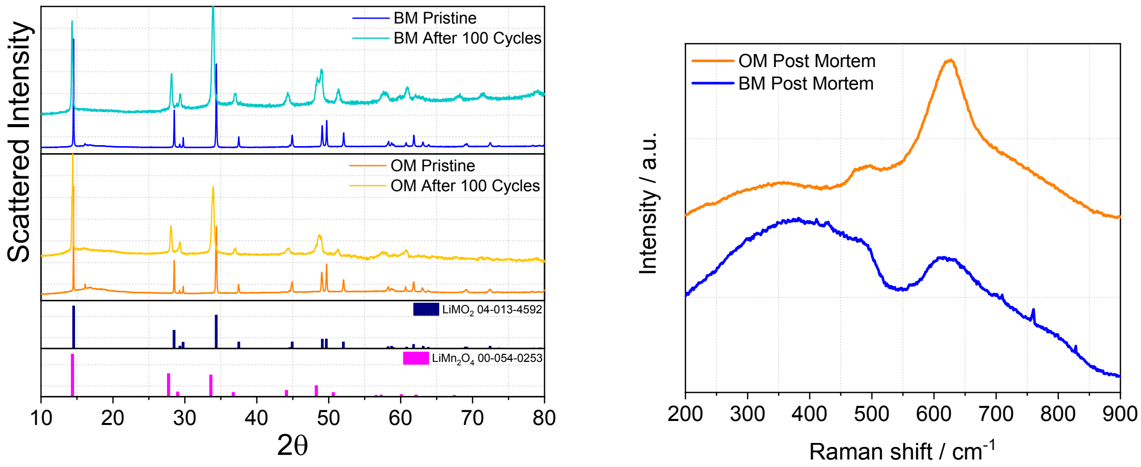

Figure S14. a)Synchrotron Diffraction pattern of BM (blue/cyano lines) and OM (orange/yellow lines) after 100 cycles in comparison with pristine ones. $LiMO_2$ and $LiMn_2O_4$ have been used as reference for layered and spinel phases. b)Post-mortem Raman spectra of BM (blue line) and OM (orange line).

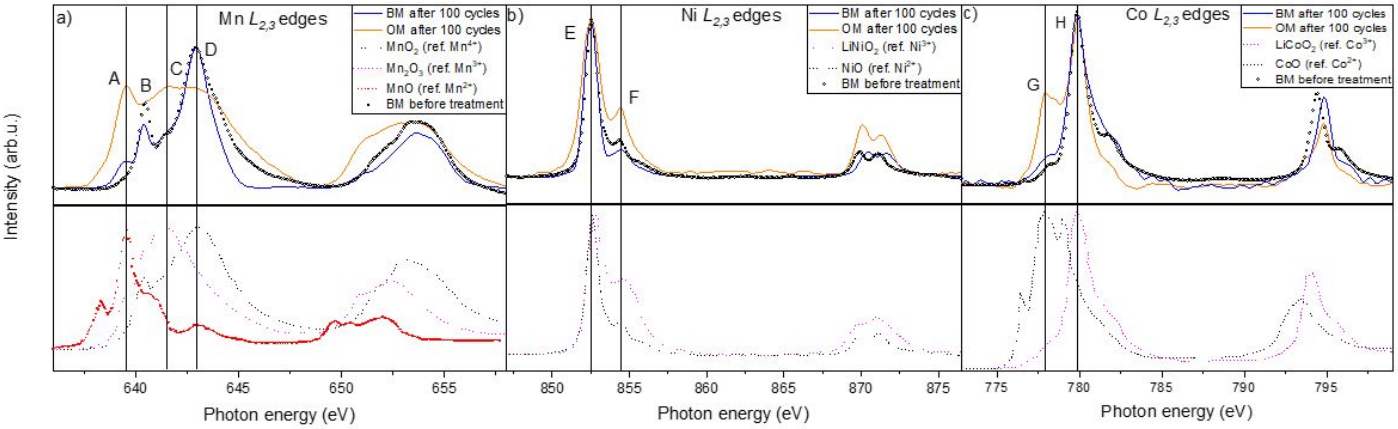

Figure S15. Upper panels: experimental $L_{2,3}$ edges XANES of a) Mn, b) Ni and c) Co measured for the BM (blue line) and OM (orange line) samples acquired after 100 cycles. For ease of comparison, the black dotted spectrum of a) b) and c) is referred to the BM sample before the charge/discharge treatment. Lower panels: experimental reference spectra for a) Mn_2O_3, MnO_2 and MnO b) NiO and $LiNiO_2$, c) $LiCoO_2$ and CoO.

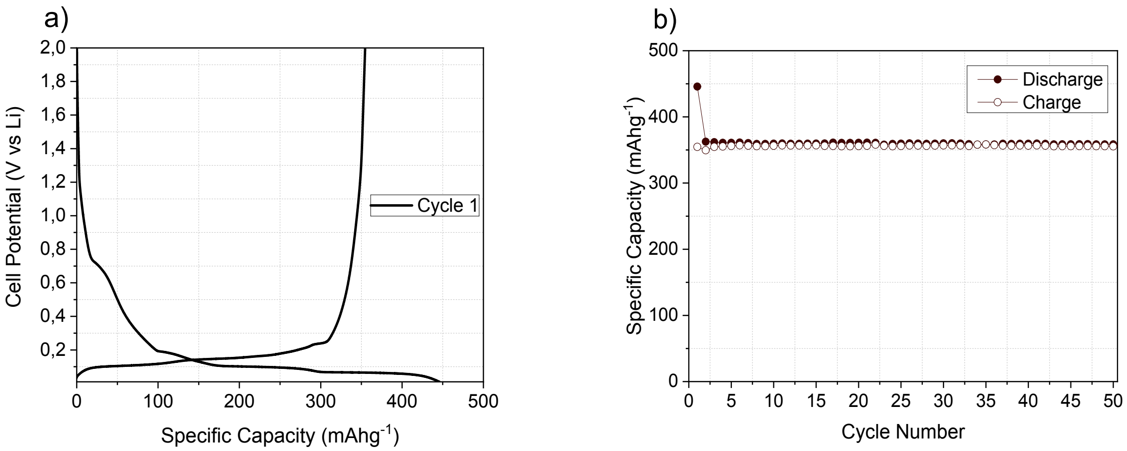

Figure S16. First cycle (a) and specific capacity vs cycle number (b) of graphite in half-cell. The current is 37.2 mA/g in a voltage range between 0.01-2V.

References

[1] B. H. Toby, Von Dreele, B. R. GSAS-II: the genesis of a modern open-source all purpose crystallography software package Journal of Applied Crystallography, 2013, 46, 544-549. DOI: doi.org/10.1107/S0021889813003531

[2] Ravel, B., Newville, ATHENA, ARTEMIS, HEPHAESTUS: data analysis for X-ray absorption spectroscopy using IFEFFIT *Journal of Synchrotron Radiation,* 2005, *12*, 537-541. DOI: 10.1107/S0909049505012719

[3] Jarvis, K. A., Deng, Z., Allard, L. F., Manthiram, A., Ferreira, P. J. Atomic structure of a lithium-rich layered oxide material for lithium-ion batteries: Evidence of a solid solution. Chemistry of Materials, 2011, 23, 3614–3621. DOI: 10.1021/cm200831c

[4] Thackeray, M. M., Kang, S. H., Johnson, C. S., Vaughey, J. T., Hackney, S. A. Comments on the structural complexity of lithium-rich $Li_{1+x}M_{1-x}O_2$ electrodes (M = Mn, Ni, Co) for lithium batteries. Electrochemistry Communications, 2006, 8, 1531–1538. DOI: 10.1016/j.elecom.2006.06.030

[5] Genevois, C., Koga, H., Croguennec, L., Ménétrier, M., Delmas, C., Weill, F. Insight into the atomic structure of cycled lithium-rich layered oxide $Li_{1.20}Mn_{0.54}Co_{0.13}Ni_{0.13}O_2$ using HAADF STEM and electron nanodiffraction. Journal of Physical Chemistry C, 2015, 119, 75–83. DOI: 10.1021/jp509388j

[6] Tuccillo, M., Palumbo, O., Pavone, M., Muñoz-García, A. B., Paolone, A., & Brutti, S. Analysis of the Phase Stability of $LiMO_2$ Layered Oxides (M= Co, Mn, Ni) Crystals, 2020, 10, 526. DOI: 10.3390/cryst10060526

[7] Kong, F., Longo, R. C., Park, M. S., Yoon, J., Yeon, D. H., Park, J. H., Cho, K. Ab initio study of doping effects on $LiMnO_2$ and Li_2MnO_3 cathode materials for Li-ion batteries. Journal of Materials Chemistry A, 2015, 3(16), 8489-8500. DOI: 10.1039/C5TA01445J

[8] Ressler, T., Brock, S. L., Wong, J., Suib, S. L. Multiple-scattering EXAFS analysis of tetraalkylammonium manganese oxide colloids. The Journal of Physical Chemistry B, 1999, 103(31), 6407-6420. DOI: 10.1021/jp9835972

[9] de Groot, F. M., Fuggle, J. C., Thole, B. T., Sawatzky, G. A. 2p x-ray absorption of 3d transition-metal compounds: An atomic multiplet description including the crystal field. Physical Review B, 1990, 42(9), 5459. DOI: 10.1103/PhysRevB.42.5459

[10] Gilbert, B., Frazer, B. H., Belz, A., Conrad, P. G., Nealson, K. H., Haskel, D., Lang, J. C., Srajer, G., De Stasio, G. Multiple scattering calculations of bonding and X-ray absorption spectroscopy of manganese oxides. The Journal of Physical Chemistry A, 2003 107(16), 2839-2847. DOI: 10.1021/jp021493s

[11] Montoro, L. A., Abbate, M., Almeida, E. C., Rosolen, J. M. Electronic structure of the transition metal ions in $LiCoO_2$, $LiNiO_2$ and $LiCo_{0.5}Ni_{0.5}O_2$. Chemical Physics Letters, 1999, 309(1-2), 14-18. DOI: 10.1016/S0009-2614(99)00650-8

[13] Di Castro, V., Polzonetti, G. XPS study of MnO oxidation. Journal of Electron Spectroscopy and Related Phenomena, 1989, 48(1), 117-123. DOI: 10.1016/0368-2048(89)80009-X
